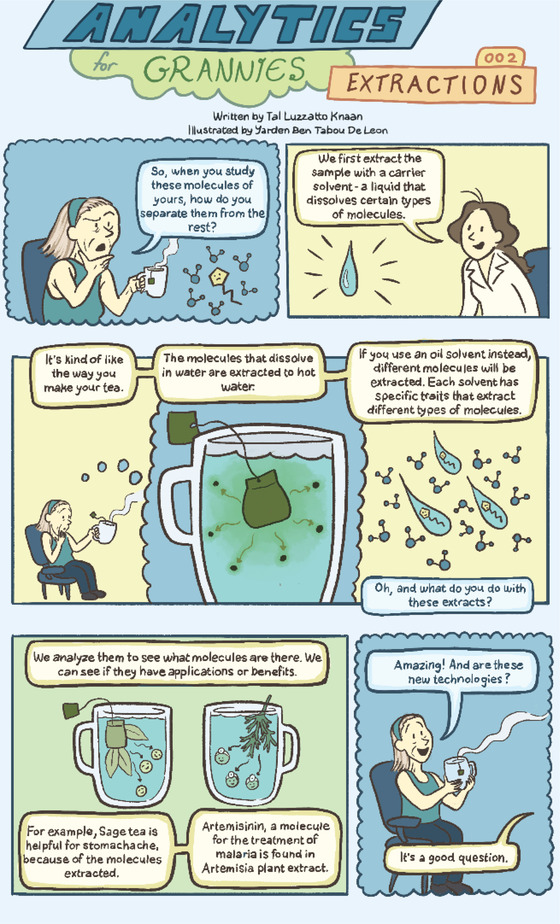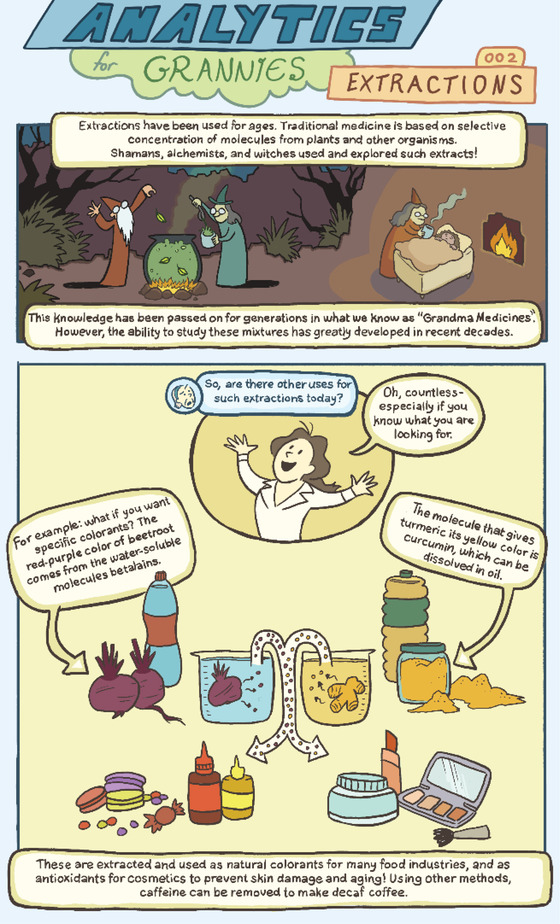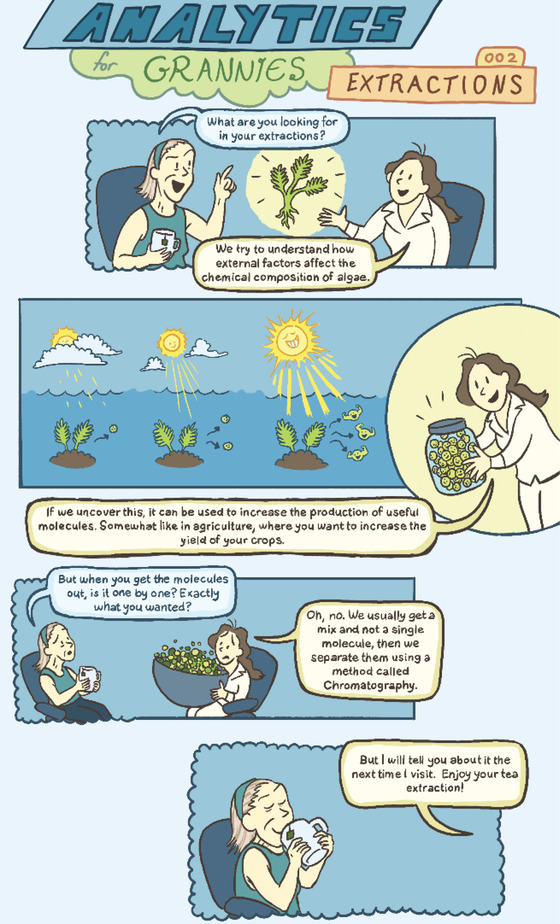# Analytics for Grannies 002: Extractions

**DOI:** 10.1002/ansa.202300900

**Published:** 2023-05-07

**Authors:** Tal Luzzatto Knaan

**Affiliations:** ^1^ Department of Marine Biology The Leon H. Charney School of Marine Sciences University of Haifa 199 Aba Koushy Ave., Mount Carmel Haifa 3498838 Israel